# Multi-Channel Spectro-Temporal Representations for Speech-Based Parkinson’s Disease Detection

**DOI:** 10.3390/jimaging11100341

**Published:** 2025-10-01

**Authors:** Hadi Sedigh Malekroodi, Nuwan Madusanka, Byeong-il Lee, Myunggi Yi

**Affiliations:** 1 Industry 4.0 Convergence Bionics Engineering, Pukyong National University, Busan 48513, Republic of Korea; hadi_sedigh@pukyong.ac.kr; 2 Digital Healthcare Research Center, College of Information Technology and Convergence, Pukyong National University, Busan 48513, Republic of Korea; nuwanv@pknu.ac.kr; 3 Major of Human Bioconvergence, Division of Smart Healthcare, Pukyong National University, Busan 48513, Republic of Korea; 4 Major of Biomedical Engineering, Division of Smart Healthcare, Pukyong National University, Busan 48513, Republic of Korea

**Keywords:** Parkinson’s Disease (PD), deep learning, speech-based diagnosis, multi-channel spectrograms, speech analysis

## Abstract

Early, non-invasive detection of Parkinson’s Disease (PD) using speech analysis offers promise for scalable screening. In this work, we propose a multi-channel spectro-temporal deep-learning approach for PD detection from sentence-level speech, a clinically relevant yet underexplored modality. We extract and fuse three complementary time–frequency representations—mel spectrogram, constant-Q transform (CQT), and gammatone spectrogram—into a three-channel input analogous to an RGB image. This fused representation is evaluated across CNNs (ResNet, DenseNet, and EfficientNet) and Vision Transformer using the PC-GITA dataset, under 10-fold subject-independent cross-validation for robust assessment. Results showed that fusion consistently improves performance over single representations across architectures. EfficientNet-B2 achieves the highest accuracy (84.39% ± 5.19%) and F1-score (84.35% ± 5.52%), outperforming recent methods using handcrafted features or pretrained models (e.g., Wav2Vec2.0, HuBERT) on the same task and dataset. Performance varies with sentence type, with emotionally salient and prosodically emphasized utterances yielding higher AUC, suggesting that richer prosody enhances discriminability. Our findings indicate that multi-channel fusion enhances sensitivity to subtle speech impairments in PD by integrating complementary spectral information. Our approach implies that multi-channel fusion could enhance the detection of discriminative acoustic biomarkers, potentially offering a more robust and effective framework for speech-based PD screening, though further validation is needed before clinical application.

## 1. Introduction

Parkinson’s Disease (PD) is the second most common neurodegenerative disorder, affecting more than 10 million individuals worldwide [1,2]. While best known for its motor symptoms, such as tremor, rigidity, and bradykinesia, PD also commonly involves speech impairments, which occur in up to 90% of patients during the course of the disease [3]. These impairments typically manifest as reduced vocal intensity, monotonic pitch, imprecise articulation, and altered speech rhythm [4,5]. Notably, such vocal changes can appear in the early stages of PD, sometimes even before motor symptoms become clinically evident. This has led to a growing interest in speech analysis as a non-invasive, accessible, and cost-effective method for early diagnosis and continuous monitoring [5]. Unlike traditional diagnostic methods that rely on clinical evaluations, neuroimaging, or subjective scales such as the Unified Parkinson’s Disease Rating Scale (UPDRS) [6,7], voice-based analysis offers the potential for objective, remote, and scalable assessment [5].

Early efforts in speech-based PD detection primarily employed handcrafted acoustic features combined with classical machine-learning (ML) classifiers [2,8]. Govindu and Palwe [9] evaluated perturbation measures such as jitter and shimmer, along with spectral descriptors, to support early diagnosis using SVM and decision tree models. Wang et al. [10] introduced Mandarin speech datasets and highlighted the utility of phonation measures for population-specific modeling. Motin et al. [11] demonstrated the feasibility of smartphone-recorded phonemes by analyzing MFCCs and energy-based parameters, highlighting the potential for mobile health applications. Mamun et al. [12] investigated vocal feature-guided ML algorithms across multiple tasks. Similarly, Hireš et al. [13] examined cross-corpus generalization of MFCCs, prosodic descriptors, and perturbation measures, revealing the challenges of deploying models across different datasets. These studies confirmed the diagnostic value of handcrafted speech features but also exposed their limitations, including sensitivity to noise, dataset dependency, and reduced ability to capture higher-level temporal dynamics.

To address these limitations, recent efforts have turned to DL, which enables models to learn hierarchical feature representations directly from raw or preprocessed audio signals [14]. This approach reduces the reliance on domain-specific feature engineering and overcomes the limitations of traditional manual feature extraction used in conventional machine-learning (ML) methods [15,16]. Elumalai et al. [17] applied long short-term memory (LSTM) networks for non-invasive PD diagnosis and severity rating, demonstrating improved robustness over shallow classifiers. Hireš et al. [15] proposed a CNN ensemble trained on spectrograms derived from sustained vowels recordings, successfully capturing complex spectro-temporal patterns in PD speech. Quan et al. [16] proposed an end-to-end deep-learning framework that extracts time-series dynamic features using time-distributed 2D-CNNs applied to mel spectrogram inputs, followed by 1D-CNNs to capture temporal dependencies, eliminating the need for handcrafted feature descriptors. Rey Paredes et al. [18] showed that CNN-based and related deep models applied directly to raw voice waveforms, combined with an augmentation strategy, significantly improve the detection of PD in a large voice dataset. Moro-Velázquez et al. [19] provided a review on articulatory and phonatory aspects, highlighting the potential of DL to capture multidimensional vocal deficits. Costantini et al. [20] compared conventional ML and deep models, emphasizing the superiority of CNNs and transformers in modeling PD-related speech changes.

More recently, self-supervised and transformer-based models have gained prominence. Vaswani et al. [21] introduced the transformer architecture, which underpins modern speech representation learning. Tougui et al. [22] applied transformer-based transfer learning on self-reported recordings, demonstrating significant improvements in generalization. Klempir et al. [23] systematically ranked pretrained embeddings, showing that Wav2Vec 2.0 outperforms earlier models across languages and tasks. In another study, Klempíř and Krupička [24] investigated Wav2Vec 1.0 embeddings for cross-database detection, while Favaro et al. [25] compared interpretable speech features with deep embeddings, noting a trade-off between accuracy and interpretability. Hemmerling et al. [26] explored vision transformers (ViTs) applied to multilingual sustained vowels. Adnan et al. [27] recently proposed a fusion model that integrates semi-supervised speech embeddings, such as Wav2Vec 2.0, WavLM, and ImageBind, achieving strong performance across diverse populations and datasets.

Despite recent advances, much of the current literature continues to rely on raw audio or a single spectro-temporal representation, typically the mel spectrogram, for speech-based PD detection. However, integrating multiple spectro-temporal views, each capturing distinct aspects of the speech signal, remains a promising yet underexplored direction. Additionally, many studies focus primarily on sustained vowel phonations, which, while useful, may not fully capture the complexity of speech motor control affected in PD. Sentence-level speech tasks, which involve prosody, articulation, and respiratory coordination, offer a richer source of potential biomarkers but have received comparatively limited attention.

In this work, we propose a multi-channel spectro-temporal approach for speech-based PD detection, leveraging sentence-level speech to capture a broader range of motor and prosodic impairments. Our method fuses complementary time–frequency representations, mel spectrogram, constant-Q transform (CQT), and gammatone spectrogram, extracted from the same speech signal. To evaluate the effectiveness of the fused multi-channel input, we assess its performance across multiple DL architectures, including convolutional and transformer-based models. Beyond classification accuracy, interpretability is essential for clinical adoption. We therefore pair t-SNE (t-distributed stochastic neighbor embedding) [28] and SHapley Additive exPlanations (SHAP) [29] to provide complementary insights. t-SNE provides an overall view of the learned embedding space, and has been used in previous PD voice studies to explore how well classes are separated and how different datasets affect high-dimensional features [30]. In parallel, SHAP (SHapley Additive exPlanations) provides local, sample-wise attributions on our fused spectrogram inputs, highlighting time–frequency regions that most influence predictions. SHAP has been used to interpret PD voice models, rank salient acoustic features [31,32,33], and more broadly, in neurological speech applications [34,35]. Together, t-SNE (global structure) and SHAP (local attributions) offer a pragmatic interpretability toolkit for speech-based PD detection, helping to contextualize learned patterns while acknowledging that clinical validation of highlighted regions remains necessary.

Overall, our results indicate that the stacked multi-channel representation consistently enhances detection performance, highlighting its potential to capture a broader range of discriminative acoustic features relevant to PD.

## 2. Materials and Methods

### 2.1. Dataset

This study used the PC-GITA dataset [36], comprising voice recordings from 100 participants, 50 diagnosed with PD and 50 healthy controls (HC). The groups were balanced by gender (25 males and 25 females per group) and age-matched (mean ages: PD = 62.2 ± 11.2, HC = 61.2 ± 11.3, range 31–86 years). PD diagnoses were confirmed by neurologists, and control participants were screened to exclude neurodegenerative disorders. Recordings were acquired in a soundproof booth at Clínica Noel, Medellín, Colombia, sampled at 44.1 kHz with 16-bit resolution, then downsampled to 16 kHz for analysis. The dataset includes several speech tasks: sustained phonation of vowels (/a/, /e/, /i/, /o/, /u/), reading 25 Spanish words, reading a dialogue, reading sentences with emphasized words, and spontaneous speech. For this study, we focused on the sentence-reading task, where each participant produced 10 repetitions of a standardized set of Spanish sentences designed to assess articulation and prosody (see Supplementary Section S1), yielding a total of 1000 audio samples. Further dataset details are available in [36].

### 2.2. Data Preprocessing

**Figure 1:**
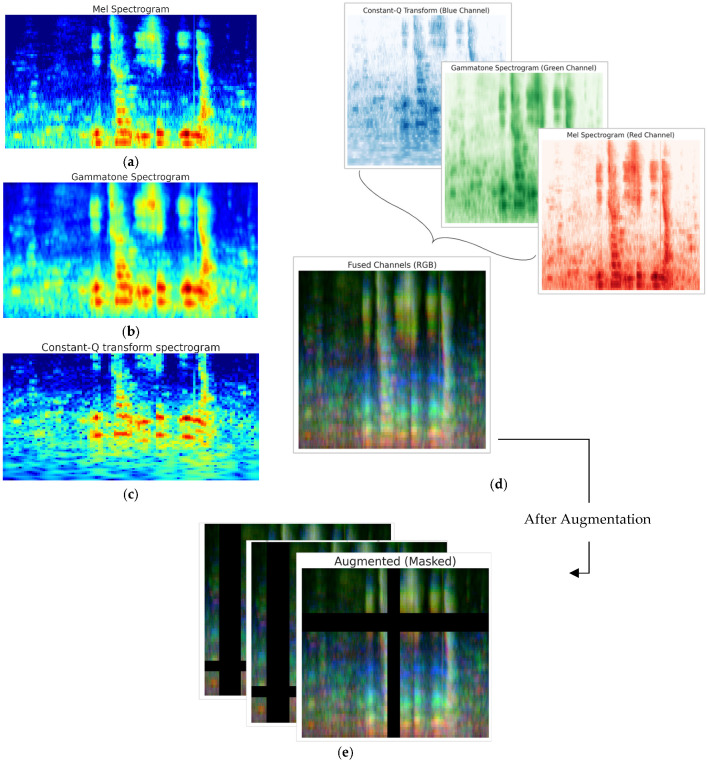
Overview of the time–frequency representations used as input features. (**a**) Mel spectrogram, (**b**) gammatone spectrogram, and (**c**) CQT are extracted from each audio clip to capture complementary spectral characteristics of speech. Each spectrogram is resized to 224 × 224 pixels and (**d**) stacked depth-wise to form a three-channel input tensor, (**e**) which is then augmented using frequency and time masking to enhance model generalization.

To enhance model robustness and generalization despite the limited dataset size, we implemented a comprehensive preprocessing and augmentation pipeline. As illustrated in Figure S1, the original audio recordings varied in length. To standardize input across samples and enable batch processing, all clips were adjusted to a fixed duration of 5 s; shorter recordings were zero-padded to preserve their temporal structure, while longer ones were truncated only after initial silence removal to minimize loss of meaningful speech content. Preprocessing included resampling all recordings to 16 kHz to reduce computational cost while preserving speech quality, peak normalization for consistent volume levels, noise reduction, and trimming of leading and trailing silences based on a 30 dB energy threshold. To further enhance the model’s ability to generalize and prevent overfitting, data augmentation techniques were applied exclusively to the training set. This choice ensures that evaluation on validation sets reflects performance on unmodified audio data. Our on-the-fly augmentation pipeline dynamically applies transformations during training, including low-pass filtering (cutoff: 150–7500 Hz), frequency masking (≤30% of frequency bins), and time masking (≤30% of time steps), effectively expanding the training dataset without requiring additional storage (Figure 1e). These techniques introduce acoustic variations that encourage robust, invariant feature learning, rather than memorization of specific training examples.

### 2.3. Multi-Channel Feature Extraction

Following preprocessing, we extracted three complementary time–frequency representations from each audio clip to capture diverse spectral characteristics of speech, as illustrated in Figure 1. The first representation is the mel spectrogram (Figure 1a), computed using 64 mel filters with a 25 ms hamming window and a 10 ms hop length. This spectrogram was log-scaled and normalized to highlight perceptually important frequency components. The second representation is the gammatone spectrogram [37] (Figure 1b), which simulates cochlear filtering through a bank of 64 filters spaced according to the equivalent rectangular bandwidth (ERB) scale [38]. The spectrogram was computed with a 25 ms frame length and 10 ms hop size, then log-scaled and normalized. The third representation is the CQT [39] (Figure 1c), extracted using 84 bins spanning seven octaves with 12 bins per octave, providing fine-grained frequency resolution, particularly in the lower frequencies where PD speech deviations often occur [40].

These representations are commonly used in speech and audio signal processing to capture features like spectral characteristics. Each spectrogram was then resized to a common dimension of 224 × 224 pixels and then stacked along the channel dimension to form a three-channel tensor, analogous to an RGB image (Figure 1d). Although structured as a three-channel ‘image’, the inputs represent acoustically distinct representations rather than spatial color information, requiring models to learn cross-representation interactions. Moreover, the size was chosen to match the input requirements of ImageNet-pretrained architectures commonly used in computer vision, enabling effective transfer learning. This fused representation, $\mathrm{Input}\;\mathrm{Tensor}\in R^{3\times 224\times 224},$ integrates complementary spectral information from the three views, thereby enhancing the model’s ability to discriminate between PD and healthy speech samples.

### 2.4. Deep-Learning Models

For the classification task, we employed a diverse set of state-of-the-art DL architectures, primarily pretrained on the ImageNet [41] dataset. These included convolutional neural networks (CNNs) such as the ResNet family (ResNet-18, 50) [42], DenseNet models (DenseNet-121, 169) [43], EfficientNet variants (EfficientNet-B0, B2) [44], and lightweight models like ShuffleNet [45] designed for efficient inference. In addition, we included standard vision transformer [46] variants. Details of the specific model variants and pretrained weights are provided in the Data Availability Statement Section. All models received as input the three-channel spectro-temporal tensor (mel spectrogram, CQT, and gammatone spectrogram), structured to match the RGB image format expected by ImageNet-pretrained models. Although originally trained on natural images, prior work has shown that low-level features from such models generalize effectively to time–frequency audio representations [13,22,47,48]. During training, all models were fully fine-tuned from their ImageNet-pretrained weights. The original classification head was replaced with a binary classifier (single-output neuron with sigmoid activation).

### 2.5. Experimental Setup

To evaluate the effectiveness of our proposed multi-channel fusion approach, we conducted a comprehensive experimental study. Our primary strategy involved a direct performance comparison between individual baseline models and our fused-input model. We began by training and testing three separate baseline models, each utilizing a single spectro-temporal representation: one with mel spectrograms, one with CQT, and one with a gammatone spectrogram. The architectures for these models were kept consistent to ensure a fair comparison. Following the baseline evaluations, we trained and tested our main model, which takes as input the stacked multi-channel data. This approach allowed us to directly quantify the performance gains achieved by combining these diverse auditory features, demonstrating the clear advantage of our fusion method for PD detection from speech signals.

To ensure a robust and unbiased evaluation, we implemented a 10-fold cross-validation protocol with subject-independent partitioning. This methodology involved dividing the dataset into ten folds, where for each iteration, the model was trained on nine folds (90% of the data) and validated on the held-out tenth fold (10% of the data). To prevent overfitting to speaker-specific characteristics and promote generalization, we enforced a strict subject-independent split. This means all recordings from a single participant were kept together within the same fold, guaranteeing that no speaker’s data appeared in both the training and validation sets in any given fold.

The experimental framework was implemented in Python (3.10.10). We utilized PyTorch (2.7.1) [49] for building and training the DL models, while data manipulation and analysis were handled with Pandas (2.3.1) [50] and NumPy (2.2.6) [51]. Model evaluation metrics and visualizations were generated using Scikit-learn (1.7.0) [52] and Matplotlib (3.10.3) [53]. The SHAP [29] library (0.48.0) was employed to interpret model predictions. The implementation is available at https://github.com/slundberg/shap (accessed on 5 August 2025).

Model training was performed using the AdamW [54] optimizer, which minimizes a cross-entropy loss function. We set an initial learning rate of 3 × 10^−4^, which was dynamically adjusted by a scheduler throughout training. Additional hyperparameters, such as batch size and the number of training epochs, are detailed in Table 1. No hyperparameter tuning was conducted; all hyperparameter values were selected based on standard practices to ensure fair comparisons across models and input types. Our goal was to isolate the effect of multi-channel fusion, leaving tuning for future work.

All experiments were conducted on a high-performance computing system equipped with an Intel Core i7 13700K CPU, 128 GB of RAM, and an NVIDIA RTX 4090 GPU with 24 GB of VRAM.

### 2.6. Evaluation Metrics

The model’s predictive performance was quantified using a suite of five evaluation criteria: accuracy, F1-score, specificity, sensitivity, and precision. The definitions for these criteria are presented in Equations (1)–(5).

(1) $$Accuracy=\frac{TP+TN}{TP+TN+FN+\;FP}$$

(2) $$Sensitivity\;(Recall)=\frac{TP}{TP+FN}$$

(3) $$Specificity=\frac{TN}{TN+FP}$$

(4) $$Precision=\frac{TP}{TP+FP}$$

(5) $$Sensitivity\;(Recall)=\frac{TP}{TP+FN}$$

where TP, TN, FP, and FN represent the counts of true positives, true negatives, false positives, and false negatives, respectively. In addition to these metrics, we also utilized an area under the receiver operating characteristic curve (AUC) and a cumulative confusion matrix across all folds to provide a comprehensive evaluation of model performance and assess overall classification accuracy.

### 2.7. Interpreting Model Predictions with SHAP

To enhance the interpretability of our PD detection model, we employed SHAP, a unified framework for explaining DL model predictions through game-theoretic principles [29]. SHAP values assign pixel-level attribution scores that quantify each time–frequency bin’s contribution to the model’s PD classification decision, enabling us to visualize which spectral regions and temporal segments of the spectrogram most significantly influence the network’s diagnostic predictions. By computing SHAP values across our dataset, we generated interpretable heatmaps that highlight the most discriminative acoustic features and spectro-temporal patterns learned by the architecture for distinguishing between PD and healthy control speech samples. This time–frequency attribution approach offers critical insights into the model’s acoustic reasoning process, highlighting spectro-temporal regions that the model finds discriminative, which may correspond to known PD-related vocal impairments such as reduced pitch variation or irregular phonation. However, without expert annotation or alignment, the clinical relevance of these regions remains inferential.

## 3. Results and Discussion

This section presents a comprehensive analysis of the results and evaluates the performance of the models examined in this study.

### 3.1. Classification Performance

To assess how different architectures and input representations perform, we evaluated EfficientNet-B2, DenseNet-121, ResNet-50, ShuffleNet, and a vision transformer Tiny using four input types: CQT, mel spectrogram, gammatone spectrogram, and their fused combination. To ensure a fair and meaningful comparison, only the best-performing variant from each deep-learning architecture family is presented in the main results (Table 2). Model selection was based strictly on the highest mean accuracy achieved with the fused multi-channel input representation. For example, within the EfficientNet family, EfficientNet-B2 (accuracy: 84.39% ± 5.19%) was selected over EfficientNet-B0 (83.09% ± 4.15%) for its superior performance. Detailed results for all evaluated models and input representations are available in the Supplementary Materials (Table S1).

Our analysis revealed a distinct performance hierarchy among both model architectures and feature representations. Notably, fused feature representations consistently outperformed individual spectral features across all architectures. This consistent improvement indicates that integrating complementary time–frequency representations captures richer acoustic information and supports more robust classification decisions.

EfficientNet-B2 emerged as the top-performing architecture overall, achieving 84.39% ± 5.19% accuracy with fused features, the highest recorded in this study. Its superior performance extends across individual representations as well, particularly with mel spectrograms (82.80% ± 8.40%). DenseNet-121 followed closely with 84.22% ± 6.15% accuracy using fused features. ResNet-50 distinguished itself by achieving the highest precision (89.51% ± 6.74%) and specificity (90.83% ± 6.18%) with fused features, suggesting that residual connections effectively minimize false positives while maintaining strong overall performance (83.75% ± 6.49% accuracy). Lightweight architectures delivered more modest, yet still competitive, results. ShuffleNet, designed for computational efficiency, achieved 80.02% ± 7.94% accuracy with fused features, while vision transformer tiny reached 83.6% ± 7.37%, demonstrating the effectiveness of attention mechanisms even in smaller-scale implementations for audio classification tasks. Additionally, we performed a brief ablation study on data augmentation, as shown in Supplementary Table S2, which demonstrated modest yet consistent improvements in model performance across different architectures. Moreover, we note that some metrics exhibit high variance, reflecting performance instability across folds, likely due to the modest dataset size and speaker variability. Despite subject-independent CV and augmentation, this underscores the need for larger, more diverse datasets.

**Figure 2:**
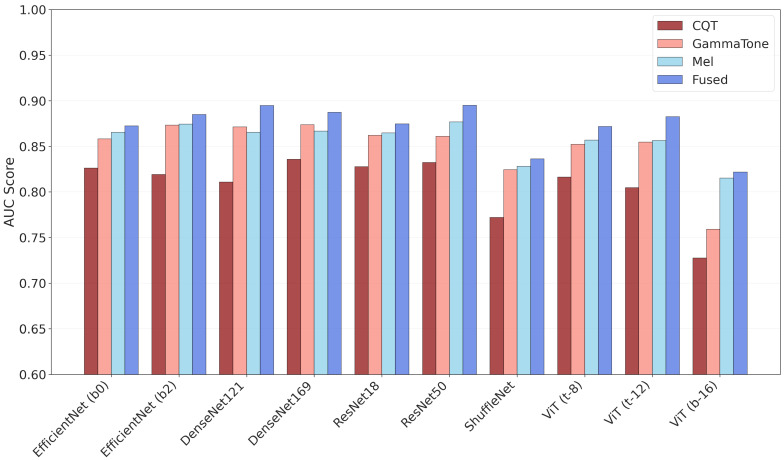
Comparison of AUC scores across various DL architectures using different audio representations: CQT, gammatone spectrogram, mel spectrogram, and their fused combination. Higher AUC values indicate better classification performance. To facilitate a clear comparison, the score scale begins at 0.60.

**Figure 3:**
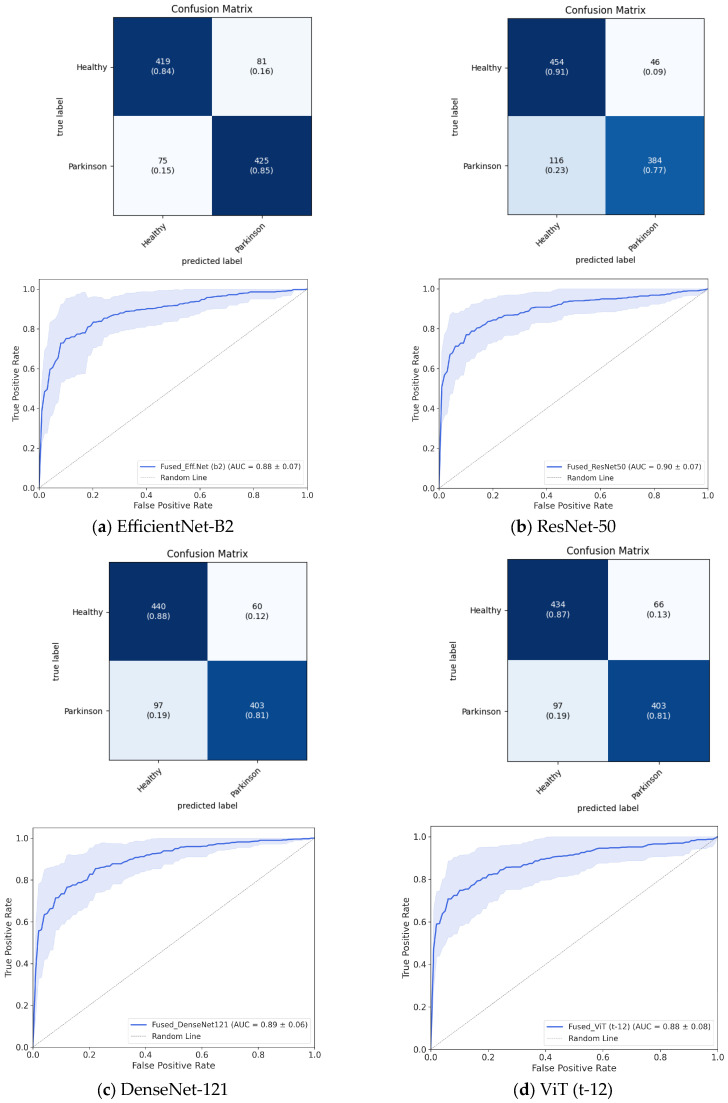
Cumulative confusion matrices and ROC curves depicting each model’s performance across cross-validation folds on the sentence dataset. (**a**) EfficientNet-B2, (**b**) ResNet-50, (**c**) DenseNet-121, and (**d**) ViT (t-12).

To visualize and further validate these performance differences, we generated a bar plot comparing AUC scores across models and representations. The bar plot (Figure 2) illustrates the AUC performance of different models across four input representations (CQT, gammatone, mel, and fused). Consistent with the tabular results, fused representations yielded the best performance across nearly all architectures, with ResNet-50 and DenseNet-121 achieving the highest mean AUC (≈0.90), indicating strong overall discriminative ability across decision thresholds. In contrast, models relying solely on CQT representations exhibited notably weaker performance, particularly with ShuffleNet and ViT (b16), where AUC values fell below 0.78. Overall, the visualization reinforces the advantage of feature fusion in enhancing classification performance and highlights the greater reliability of DenseNet, ResNet, and EfficientNet models when using fused inputs. Complementary analyses for the best-performing models (Figure 3), including cumulative confusion matrices and ROC curves, further corroborate these findings. Specifically, while EfficientNet-B2 achieves a balanced sensitivity–specificity profile, Dense-Net-121 and ResNet-50 attain comparable discriminative power (AUC ≈ 0.90), with marginally higher precision and specificity as noted earlier.

Moreover, to evaluate potential demographic bias, we conducted a fairness analysis across gender and age subgroups using our top three models (DenseNet-121, ResNet-50, and EfficientNet-B2). Evaluating 1000 samples evenly split by gender (500 female, 500 male) and age (≤62 vs. >62 years, based on median), we found no statistically significant performance differences (*p* > 0.05, independent *t*-test) in accuracy across groups (Table 3).

**Figure 4:**
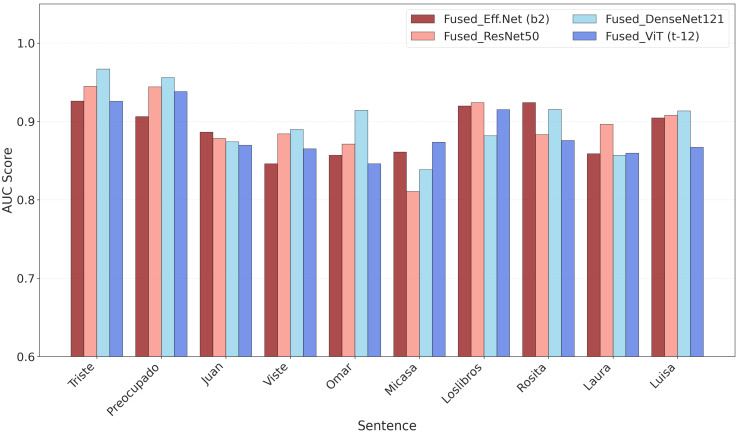
Mean AUC scores across models for 10 sentence types. Emphasized and emotionally salient sentences (e.g., “Triste”, “Juan”) achieve higher accuracy, while syntactically simple, neutral sentences (e.g., “Mi casa”) show lower performance, highlighting the role of prosody and affect in classification.

To explore how sentence content influences detection and to assess model performance across diverse contexts, we measured AUC scores for each of the 10 sentence types (Figure 4), encompassing both syntactically simple and complex utterances, as well as emotionally expressive sentences marked by emphasized prosody. The sentences were drawn from two task categories: Sentence Repetition (varying in syntactic complexity) and emphasized Sentence Reading (featuring stress on key words). The AUC values reflect each model’s ability to discriminate between PD and HC speech samples from these utterances.

The results reveal systematic variation in AUC performance across sentence types, suggesting that acoustic and linguistic characteristics modulate model discriminability. Notably, sentences with emotionally charged or prosodically emphasized content consistently achieved higher AUC values across all models. For example, “Triste” (Estoy muy triste…) and “Preocupado” (Estoy muy preocupado…), both expressing negative affect and featuring prominent intonational contours, yielded among the highest AUC scores (≥0.93), with minimal inter-model variance. Similarly, the prosodically marked sentences “Juan” (ROMPIÓ una PIERNA…) and “Viste” (GANAR la medalla…) demonstrated strong detection performance (AUC > 0.87), indicating that exaggerated stress patterns, particularly on verbs denoting physical events or outcomes, enhance signal detectability.

In contrast, syntactically simple and emotionally neutral utterances, such as “Mi casa” (Mi casa tiene tres cuartos), exhibited comparatively lower AUC scores (ranging from ~0.81 to 0.86), particularly in ResNet-50. This can suggest that minimal syntactic structure and low emotional valence may reduce the acoustic footprint of PD-related dysprosody, thereby diminishing model sensitivity. However, syntactic complexity alone does not fully explain performance differences. Sentences like “Omar”, “Laura”, and “Rosita”, which contain relative clauses (e.g., que vive cerca, que pinta bien), showed elevated AUC values despite moderate syntactic load, likely due to increased phonetic duration, pause placement, and contextual richness that provide additional temporal and spectral cues for classification. Interestingly, the sentence “Los libros” (Los libros nuevos no caben…), though syntactically simple, performed well, suggesting that lexical content and semantic context, such as negation and spatial constraints, may also contribute to discriminability. Furthermore, “Luisa” (compra el colchón duro que tanto le gusta), which combines syntactic embedding with a declarative tone, achieved high performance, reinforcing the role of structured discourse in enhancing acoustic variability.

These findings suggest that sentence-level acoustic characteristics, particularly those associated with prosodic emphasis and emotional expression, may enhance the detectability of speech impairments in PD. While syntactic structure appears to contribute, the most consistent performance gains were observed in utterances with increased acoustic prominence, which may amplify PD-related dysprosody. Nevertheless, this analysis remains exploratory, and further linguistic and clinical investigation is needed to disentangle the respective roles of syntax, prosody, and emotion on model performance.

To benchmark our method against prior work, we compared our results with recent studies utilizing the PC-GITA dataset’s sentence reading task. Prior research has applied both ML and DL approaches to this dataset, with particular emphasis on sentence reading due to its controlled and linguistically consistent speech samples across subjects. In this study, we further contextualize our performance by comparing it with these works, as summarized in Table 4. Using the sentence reading subset of PC-GITA and a multi-channel spectrogram representation with EfficientNet-B2, our approach achieves an F1-score of 84.35 ± 5.52%. The reported variability across folds is within the range typically seen in PD speech studies on this dataset (≈5–10%), reflecting differences in speaker partitions but still indicating stable and generalizable performance. This performance is competitive with the previous methods, including handcrafted acoustic features (e.g., MFCCs) combined with XGBoost classification (Favaro et al. [25], 57%), and foundation speech recognition models like Wav2Vec (82%), WavLM (81.99%), and HuBERT (80.84%) trained on raw audio [25,55,56,57]. While direct comparison is limited by differences in model architecture, pretraining strategy, and input representation, our results suggest that carefully engineered multi-channel spectro-temporal inputs can achieve competitive performance for PD detection without relying on large-scale audio pretraining.

### 3.2. SHAP and t-SNE Feature Visualization

**Figure 5:**
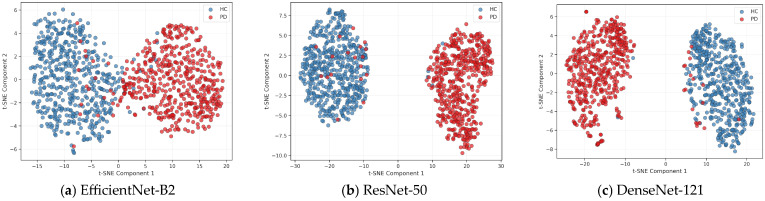
Comparison of t-SNE embeddings for HC and PD classes across the three best-performing DL architectures, (**a**) EfficientNet-B2, (**b**) ResNet-50, and (**c**) DenseNet-121, revealing distinct clustering patterns based on true labels.

To gain insights into the learned representations and decision-making processes of our models, we integrated global and local interpretability methods (t-SNE and SHAP) in this subsection. For consistency, both visualizations are based on the model trained on the first fold of cross-validation, ensuring directly comparable analyses. This approach ensures that both interpretability methods are based on the same learned representation, enabling a direct and comparable analysis of feature importance and embedding structure across different architectures. The t-SNE visualizations presented in Figure 5 illustrate the separation of high-dimensional feature representations learned by three deep convolutional neural networks that have shown better performance (EfficientNet-B2, ResNet-50, and DenseNet-121) when applied to a classification task distinguishing between HC (blue) and PD (red) subjects. Each plot projects the embedding into a two-dimensional space, revealing how each model captures the underlying structure of the data. EfficientNet-B2 exhibits a moderate degree of class separation with some overlap, suggesting that while it captures meaningful features, certain regions remain ambiguous. In contrast, ResNet-50 and DenseNet-121 demonstrate a clearer distinction between HC and PD clusters with minimal inter-class overlap, indicating stronger discriminative capabilities in the learned feature space. Since t-SNE is a nonlinear 2D projection, it may distort high-dimensional structure. EfficientNet-B2 might rely on more distributed features that appear less separable in this space yet still contribute to its higher accuracy. Meanwhile, the somewhat clearer clusters and slightly higher AUC observed for ResNet-50 and DenseNet-121 suggest these models may also serve as competitive alternatives. Importantly, visualizations here are based on Fold 1 for consistency with SHAP; projections for more folds are provided in Supplementary Figure S2 to assess stability. Nonetheless, all 2D projections should be interpreted with caution, as nonlinear dimensionality reduction can introduce artifacts.

**Figure 6:**
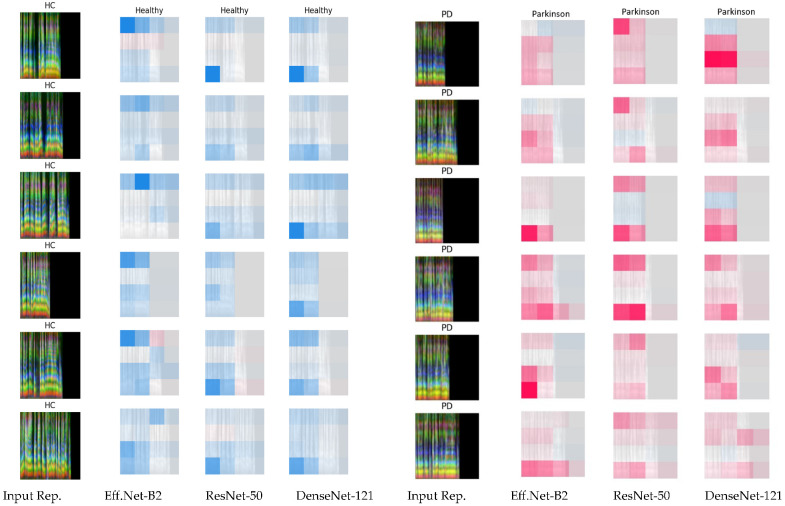
SHAP heatmaps using fused spectrograms for PD detection across EfficientNet-B2, ResNet-50, and DenseNet-121, with blue indicating contributions to “Healthy” predictions and red indicating contributions to “Parkinson” predictions. The left column displays fused voice spectrograms from HC and PD patients; adjacent plots highlight model-relevant features that drive classification decisions.

To further investigate the features driving the classification decisions of these models, we applied SHAP, a game-theoretic approach that interprets individual predictions by attributing importance scores to input pixels. Unlike t-SNE, which provides a global view of the learned embedding structure, SHAP offers local interpretability, revealing which regions of the input image most influence the model’s decision for a given sample. Figure 6 presents SHAP heatmaps that depict the feature contributions of fused spectrograms in differentiating between healthy controls (HC) and patients with PD. Additional examples from different folds can be found in Supplementary Materials Figure S3. Each row corresponds to a sample: the leftmost column displays the input fused spectrogram, while the adjacent panels show SHAP attribution maps from three DL models, EfficientNet-B2, ResNet-50, and DenseNet-121, highlighting regions influential in classification decisions. The selected samples include the utterance “Viste,” which consistently achieved high classification performance across all models, as demonstrated in Figure 4. In the SHAP visualizations, negative values (displayed in blue) indicate regions that contribute to a prediction of HC, whereas positive values (shown in red) reflect features that support a PD classification.

For HC samples, the SHAP heatmaps predominantly exhibit blue regions across all models, indicating that specific spectral patterns contribute positively to “Healthy” predictions. These patterns demonstrate a consistent focus on specific time–frequency regions, particularly in the lower-frequency bands and temporal segments of the spectrogram. This aligns with clinical observations that PD affects fundamental frequency control and laryngeal stability, particularly in lower-frequency bands [40,58,59], though further validation with expert annotation is required. In contrast, PD samples are characterized by dominant red regions in the heatmaps, signifying features that drive “Parkinson” classifications. These red activations are more widespread and intense, especially in the lower- and upper-frequency ranges as well as in later time segments, suggesting that the models capture distinct temporal and spectral deviations associated with PD. EfficientNet-B2 demonstrates a more uniform and localized activation pattern, whereas ResNet-50 and DenseNet-121 generate broader but less consistent heatmaps.

Finally, while the SHAP heatmaps highlight discriminative acoustic regions, their clinical meaning remains uncertain without expert input. Future work should involve close collaboration with speech-language pathologists and neurologists to validate whether these highlighted patterns correspond to established clinical features of Parkinson’s-related speech impairments. Such integration would help ensure that explainability outcomes contribute meaningfully to clinical practice.

## 4. Conclusions

In conclusion, our multi-channel spectro-temporal approach, which fuses mel spectrogram, CQT, and gammatone representations, demonstrates improved performance in detecting PD from sentence-level speech. By integrating complementary acoustic features, the method enhances the ability of DL models to capture subtle speech impairments associated with PD. Results across multiple architectures, particularly EfficientNet-B2, consistently show gains from feature fusion, achieving up to 84.39% classification accuracy and outperforming both single-channel inputs and prior approaches on the same dataset. Interpretability analyses using SHAP suggest that models leverage frequency and temporal patterns, although the clinical relevance of these features remains to be validated. Complementary t-SNE visualizations tentatively show improved class separation in deeper CNN networks. While these findings are encouraging, validation on larger and more heterogeneous cohorts, including recordings from real-world, noisy environments, is needed to ensure generalizability and clinical viability. Future work should also involve clinical experts to assess the interpretability outputs, thereby strengthening the translational value of explainable AI in speech-based PD screening. Taken together, this study highlights the potential of integrating complementary spectro-temporal representations with DL for non-invasive, speech-based screening.

## Figures and Tables

**Table 1:** Hyperparameter settings for training models.

Hyperparameter	Values
Epochs	50
Batch size	16
Initial Learning Rate	3 × 10^−4^
Optimizer	AdamW (β_1_ = 0.9, β_2_ = 0.999, Weight decay = 0.01)
Scheduler	Cosine annealing scheduler with initial warm-up
Loss	Cross-entropy loss

**Table 2:** Summary of classification performance (mean ± standard deviation) for each model across different input representations. Only the best result from each model architecture family is reported. Full results are provided in the Supplementary Materials (Table S1). Boldface indicates the best score for each metric across all models, while underlined values denote the second-best.

Model	Input Rep. ^a^	Accuracy	F1-Score	Sensitivity	Precision	Specificity	AUC
Eff.Net-B2	CQT	0.772 ± 0.086	0.770 ± 0.085	0.768 ± 0.126	0.787 ± 0.108	0.778 ± 0.126	0.819 ± 0.094
	Mel	0.828 ± 0.084	0.825 ± 0.089	0.828 ± 0.145	0.837 ± 0.084	0.831 ± 0.112	0.874 ± 0.094
	Gamma	0.822 ± 0.074	0.806 ± 0.092	0.766 ± 0.156	0.876 ± 0.079	0.881 ± 0.084	0.873 ± 0.075
	Fused	**0.843 ± 0.051**	**0.843 ± 0.055**	**0.850 ± 0.098**	0.845 ± 0.066	0.838 ± 0.081	0.884 ± 0.073
DenseNet-121	CQT	0.777 ± 0.084	0.781 ± 0.087	0.806 ± 0.131	0.768 ± 0.084	0.748 ± 0.112	0.810 ± 0.116
	Mel	0.830 ± 0.072	0.819 ± 0.087	0.798 ± 0.151	0.868 ± 0.083	0.864 ± 0.118	0.865 ± 0.106
	Gamma	0.818 ± 0.068	0.808 ± 0.077	0.778 ± 0.121	0.853 ± 0.072	0.861 ± 0.080	0.871 ± 0.075
	Fused	0.842 ± 0.061	0.831 ± 0.077	0.806 ± 0.148	0.881 ± 0.075	0.883 ± 0.091	0.894 ± 0.063
ResNet-50	CQT	0.784 ± 0.083	0.764 ± 0.111	0.736 ± 0.179	0.824 ± 0.090	0.836 ± 0.094	0.832 ± 0.091
	Mel	0.799 ± 0.067	0.783 ± 0.092	0.764 ± 0.183	0.844 ± 0.091	0.839 ± 0.120	0.876 ± 0.095
	Gamma	0.817 ± 0.073	0.807 ± 0.083	0.778 ± 0.128	0.854 ± 0.079	0.858 ± 0.089	0.861 ± 0.086
	Fused	0.837 ± 0.064	0.823 ± 0.072	0.768 ± 0.099	**0.895 ± 0.067**	**0.908 ± 0.061**	**0.895 ± 0.074**
ShuffleNet	CQT	0.727 ± 0.072	0.707 ± 0.092	0.676 ± 0.133	0.753 ± 0.065	0.774 ± 0.078	0.772 ± 0.091
	Mel	0.790 ± 0.082	0.774 ± 0.098	0.750 ± 0.168	0.824 ± 0.080	0.833 ± 0.094	0.828 ± 0.106
	Gamma	0.771 ± 0.066	0.755 ± 0.076	0.718 ± 0.133	0.822 ± 0.109	0.824 ± 0.123	0.824 ± 0.065
	Fused	0.800 ± 0.079	0.790 ± 0.089	0.774 ± 0.152	0.831 ± 0.104	0.829 ± 0.122	0.836 ± 0.087
ViT (t)	CQT	0.751 ± 0.086	0.743 ± 0.088	0.720 ± 0.105	0.776 ± 0.096	0.782 ± 0.119	0.804 ± 0.106
	Mel	0.789 ± 0.080	0.768 ± 0.114	0.738 ± 0.183	0.839 ± 0.084	0.842 ± 0.136	0.856 ± 0.095
	Gamma	0.818 ± 0.085	0.800 ± 0.110	0.764 ± 0.173	0.865 ± 0.063	0.876 ± 0.065	0.854 ± 0.103
	Fused	0.836 ± 0.073	0.828 ± 0.081	0.806 ± 0.134	0.867 ± 0.080	0.867 ± 0.100	0.882 ± 0.087

^a^ Input representations: CQT spectrogram, gammatone spectrogram, mel spectrogram, and a fused representation obtained by stacking channels.

**Table 3:** Statistical analysis across demographic subgroups (gender and age).

Demographic Property	Model	Group	Number of Samples	Group Accuracy	*p*-Value	Significant?
Gender	DenseNet-121	Male	500	0.8420	0.9037	No
		Female	500	0.8440		
	ResNet-50	Male	500	0.8080	0.0993	No
		Female	500	0.8680		
	EfficientNet-B2	Male	500	0.8260	0.3845	No
		Female	500	0.8520		
Age	DenseNet-121	Above 62	530	0.8532	0.5275	No
		62 and below	470	0.8340		
	ResNet-50	Above 62	530	0.8787	0.1197	No
		62 and below	470	0.8019		
	EfficientNet-B2	Above 62	530	0.8553	0.4260	No
		62 and below	470	0.8245		

**Table 4:** Performance comparison of recent speech-based PD detection methods using the same dataset (sentence reading task, PC-GITA).

Study	F1-Score [%]	Approach
A. Favaro et al. [25]	57% ^a^	XGBoost on prosodic, linguistic, and cognitive handcrafted descriptors.
A. Favaro et al. [25]	82% ^a^	Wav2Vec2.0/HuBERT/TRILLsson embeddings from raw audio; pooled + simple classifier.
N. Narendra et al. [55]	68.52 ± 8.85	1D-CNN on raw glottal flow (QCP inverse filtering); compared to SVM on handcrafted features.
M. L. Quatra et al. [56]	81.99 ± 8.34	WavLM base fine-tuned; layer-weighted sum + attention pooling.
M. L. Quatra et al. [56]	80.84 ± 9.17	HuBERT base, with the same setup, combined with WavLM for optimal performance.
T. Yi Zhong et al. [57]	74.19 ± 10.80	WavLM base fine-tuned; attention pooling; tested on conversational (TT) vs. clinical (PC-GITA) data.
Current Study	84.35 ± 5.52	EfficientNet-B2, using multi-channel spectrograms.

^a^ Standard deviation was not reported in the original study.

## Data Availability

The dataset used in this study is not publicly available but can be obtained upon reasonable request from Juan Rafael Orozco-Arroyave, affiliated with the Universidad de Antioquia (UdeA). The pre-trained models used in this study include EfficientNet-B0 and B1, ResNet-18 and ResNet-50, DenseNet-121 and DenseNet-169, and ShuffleNetV2, all of which are available via PyTorch Hub. Additionally, vision transformer (ViT) such as ViT-Tiny (t-8 and t-12) and ViT-Base (b-16) were utilized, available through the Hugging Face timm library. The source code is also available on reasonable request.

## Supplementary Materials

The following supporting information can be downloaded at https://www.mdpi.com/article/10.3390/jimaging11100341/s1: Section S1: Details on sentence reading task; Figure S1: Histogram showing the distribution of audio lengths for the sentence reading across the groups: HC and PD; Table S1: Summary of classification performance (mean ± standard deviation) for each model across different input representations; Table S2: Performance comparison of different models with and without data augmentation; Figure S2: Comparison of t-SNE embeddings for HC and PD classes across three best-performing deep-learning architectures across different folds; Figure S3: SHAP heatmaps across folds for PD detection using fused spectrograms.

## Abbreviations

The following abbreviations are used in this manuscript:

PD	Parkinson’s Disease
HC	Healthy Controls
CQT	Constant-Q Transform
CNN	Convolutional Neural Network
ViT	Vision Transformer
SHAP	SHapley Additive exPlanations
AUC	Area Under the ROC Curve
ROC	Receiver Operating Characteristic
TP	True Positive
TN	True Negative
FP	False Positive
FN	False Negative
ERB	Equivalent Rectangular Bandwidth
F1	F1-Score
PC-GITA	Parkinson’s Spanish Speech Corpus
RGB	Red–Green–Blue
t-SNE	t-Distributed Stochastic Neighbor Embedding
AdamW	Adaptive Moment Estimation with Weight Decay
dB	Decibel
UPDRS	Unified Parkinson’s Disease Rating Scale
Mel	Mel spectrogram (used contextually)
Gamma	Gammatone Spectrogram (used contextually)
