# Supplementary material for: Multi-Channel Spectro-Temporal Representations for Speech-Based Parkinson’s Disease Detection
Source: J Imaging. 2025 Oct 1;11(10):341. doi: 10.3390/jimaging11100341 (PMC12565443; doi:10.3390/jimaging11100341)
Supplement: Supplementary file 1 [file jimaging-11-00341-s001.zip › jimaging-3872784-supplementary.pdf]

Multi-Channel Spectro-Temporal Representations for Speech-Based Parkinson's Disease Detection

Hadi Sedigh Malekroodi [1], Nuwan Madusanka [2], Byeong-il Lee [1,2,3,*] and Myunggi Yi [1,2,4,*]

[1] Industry 4.0 Convergence Bionics Engineering, Pukyong National University, Busan 48513, Republic of Korea; hadi_sedigh@pukyong.ac.kr
[2] Digital Healthcare Research Center, College of Information Technology and Convergence, Pukyong National University, Busan 48513, Republic of Korea; nuwanv@pknu.ac.kr
[3] Major of Human Bioconvergence, Division of Smart Healthcare, Pukyong National University, Busan 48513, Republic of Korea
[4] Major of Biomedical Engineering, Division of Smart Healthcare, Pukyong National University, Busan 48513, Republic of Korea
* Correspondence: bilee@pknu.ac.kr (B.-i.L.); myunggi@pknu.ac.kr (M.Y.)

Supplementary materials include:

Section S1: Details on Sentence reading Task.

Figure S1. Histogram showing the distribution of audio lengths for the sentence reading across the groups: HC and PD.

Table S1. Summary of classification performance (mean ± standard deviation) for each model across different input representations.

Table S2. Performance comparison of different models with and without data augmentation.

Figure S2. Comparison of t-SNE embeddings for HC and PD classes across three best-performing deep learning architectures across different folds.

Figure S3. SHAP heatmaps across folds for PD detection using fused spectrograms.

Section S1

1) Sentence Repetition: Simple vs. Complex Syntax [1]

As part of the speech tasks, participants were asked to repeat a set of sentences designed to vary in syntactic complexity. The list below includes both the original Spanish sentences and their English translations, categorized as either simple or complex:

(a) *Mi casa tiene tres cuartos.* (Simple)
English: My house has three rooms.

(b) *Omar, que vive cerca, trajo miel.* (Complex)
English: Omar, who lives nearby, brought honey.

(c) *Laura sube al tren que pasa.* (Complex)
English: Laura gets on the train that is passing by.

(d) *Los libros nuevos no caben en la mesa de la oficina.* (Simple)
English: The new books don't fit on the office table.

(e) *Rosita Niño, que pinta bien, donó sus cuadros ayer.* (Complex)
English: Rosita Niño, who is a good painter, donated her artworks yesterday.

(f) *Luisa Rey compra el colchón duro que tanto le gusta.* (Complex)
English: Luisa Rey buys the firm mattress that she likes so much.

2) Emphasized Sentence Reading [1]

Another task involved reading sentences with intentional stress on specific words, indicated by capital letters. These samples aim to reflect expressive prosody. Below are the emphasized Spanish sentences and their English equivalents:

(a) *Viste las noticias? Yo vi GANAR la medalla de plata en pesas. Ese muchacho tiene mucha fuerza!*
English: Did you see the news? I saw him WIN the silver medal in weightlifting. That guy is really strong!

(b) *Juan se ROMPIÓ una PIERNA cuando iba en la MOTO.*
English: Juan BROKE his LEG while riding the MOTORCYCLE.

(c) *Estoy muy triste, ayer vi MORIR a un amigo.*
English: I'm very sad — yesterday I saw a friend DIE.

(d) *Estoy muy preocupado, cada vez me es más difícil HABLAR.*
English: I'm very worried — it's getting harder and harder to TALK.

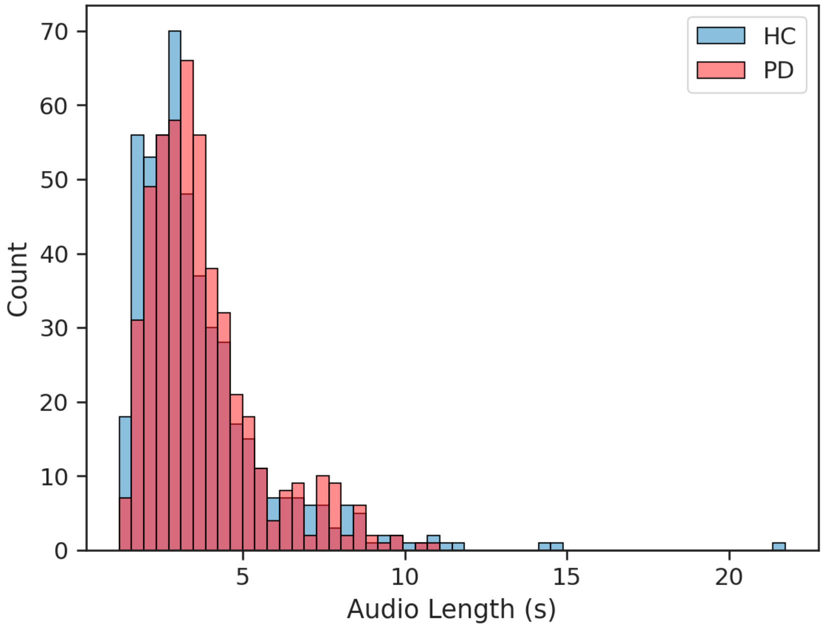

Figure S1. Histogram showing the distribution of audio lengths for the sentence reading across the groups: HC and PD.

Table S1. Summary of classification performance (mean ± standard deviation) for each model across different input representations.

Model	Input Rep.	Accuracy	F1-Score	Sensitivity	Precision	Specificity	AUC
**efficientnet (b0)**	CQT	0.7925 ± 0.0782	0.7919 ± 0.0789	0.796 ± 0.1142	0.7968 ± 0.0858	0.7928 ± 0.0974	0.8263 ± 0.0975
	Mel	0.8191 ± 0.0942	0.8143 ± 0.1042	0.814 ± 0.1572	0.8308 ± 0.1021	0.8282 ± 0.1139	0.8655 ± 0.0989
	Gamma	0.8077 ± 0.0779	0.8041 ± 0.089	0.806 ± 0.1445	0.8205 ± 0.0877	0.8073 ± 0.1246	0.8583 ± 0.0861
	Fused	0.8309 ± 0.0415	0.8243 ± 0.0498	0.802 ± 0.0916	0.8558 ± 0.0512	0.8608 ± 0.0691	0.8723 ± 0.0656
**efficientnet (b2)**	CQT	0.7725 ± 0.0861	0.7702 ± 0.0853	0.768 ± 0.1262	0.7872 ± 0.1083	0.7783 ± 0.1265	0.8193 ± 0.0949
	Mel	0.828 ± 0.084	0.8252 ± 0.0892	0.828 ± 0.1458	0.837 ± 0.0841	0.8315 ± 0.1122	0.8743 ± 0.0942
	Gamma	0.8229 ± 0.0742	0.8062 ± 0.0922	0.766 ± 0.1569	0.8766 ± 0.079	0.8818 ± 0.0846	0.8733 ± 0.0756
	Fused	0.8439 ± 0.0519	0.8435 ± 0.0552	0.85 ± 0.0985	0.8458 ± 0.0666	0.838 ± 0.0819	0.8849 ± 0.0738
**densenet121**	CQT	0.7776 ± 0.0843	0.7813 ± 0.0874	0.806 ± 0.1313	0.7685 ± 0.0848	0.7483 ± 0.1129	0.8109 ± 0.1162
	Mel	0.8303 ± 0.0727	0.8198 ± 0.0879	0.798 ± 0.1513	0.8689 ± 0.0836	0.8642 ± 0.1187	0.8655 ± 0.1068
	Gamma	0.8188 ± 0.0687	0.8089 ± 0.0776	0.778 ± 0.1213	0.8538 ± 0.0721	0.8617 ± 0.0805	0.8713 ± 0.075
	Fused	0.8422 ± 0.0615	0.8317 ± 0.0773	0.806 ± 0.1485	0.8819 ± 0.075	0.8838 ± 0.0918	0.8948 ± 0.0633
**densenet169**	CQT	0.7964 ± 0.0851	0.7977 ± 0.0818	0.808 ± 0.1367	0.8061 ± 0.0972	0.7872 ± 0.1469	0.8358 ± 0.098
	Mel	0.8118 ± 0.0684	0.8038 ± 0.0834	0.794 ± 0.1558	0.8398 ± 0.081	0.83 ± 0.128	0.8669 ± 0.09
	Gamma	0.8341 ± 0.0775	0.8204 ± 0.0945	0.786 ± 0.1552	0.875 ± 0.048	0.8837 ± 0.0564	0.8736 ± 0.086
	Fused	0.8403 ± 0.077	0.8267 ± 0.0961	0.794 ± 0.1473	0.8766 ± 0.0651	0.8893 ± 0.0623	0.8873 ± 0.0885
**resnet18**	CQT	0.7867 ± 0.0845	0.7821 ± 0.0916	0.778 ± 0.1328	0.8001 ± 0.0904	0.7965 ± 0.1189	0.8278 ± 0.0951
	Mel	0.8279 ± 0.0607	0.8215 ± 0.0686	0.808 ± 0.1186	0.8474 ± 0.0715	0.849 ± 0.0737	0.8649 ± 0.0913
	Gamma	0.817 ± 0.0917	0.7982 ± 0.1133	0.754 ± 0.1619	0.8691 ± 0.0799	0.8828 ± 0.0879	0.8623 ± 0.0964
	Fused	0.8294 ± 0.068	0.818 ± 0.0797	0.79 ± 0.1461	0.8685 ± 0.0763	0.8727 ± 0.0975	0.8746 ± 0.1022
**resnet50**	CQT	0.7844 ± 0.0839	0.7646 ± 0.1111	0.736 ± 0.1796	0.8247 ± 0.09	0.8368 ± 0.094	0.8322 ± 0.0913
	Mel	0.7997 ± 0.0676	0.7839 ± 0.0926	0.764 ± 0.1835	0.8447 ± 0.0911	0.839 ± 0.1208	0.8768 ± 0.0954
	Gamma	0.8174 ± 0.073	0.8073 ± 0.0834	0.778 ± 0.1284	0.8545 ± 0.0799	0.8587 ± 0.0899	0.8611 ± 0.0865
	Fused	0.8375 ± 0.0649	0.8237 ± 0.0729	0.768 ± 0.0999	0.8951 ± 0.0674	0.9083 ± 0.0618	0.8951 ± 0.0747
**shufflenet**	CQT	0.7271 ± 0.0726	0.7073 ± 0.092	0.676 ± 0.1333	0.7533 ± 0.0656	0.7745 ± 0.078	0.772 ± 0.0917
	Mel	0.7901 ± 0.082	0.7748 ± 0.098	0.75 ± 0.1685	0.8243 ± 0.0809	0.8332 ± 0.0942	0.8282 ± 0.106
	Gamma	0.7711 ± 0.0665	0.7553 ± 0.076	0.718 ± 0.1331	0.8222 ± 0.1091	0.8243 ± 0.1231	0.8244 ± 0.0656
	Fused	0.8002 ± 0.0794	0.7903 ± 0.0899	0.774 ± 0.1523	0.8319 ± 0.1049	0.829 ± 0.1228	0.8361 ± 0.0877
**ViT (t-8)**	CQT	0.7658 ± 0.0704	0.758 ± 0.0771	0.742 ± 0.1133	0.7831 ± 0.0782	0.7903 ± 0.0855	0.8164 ± 0.0869
	Mel	0.7976 ± 0.074	0.7803 ± 0.0923	0.744 ± 0.1543	0.8431 ± 0.0897	0.856 ± 0.0868	0.8569 ± 0.0938
	Gamma	0.8089 ± 0.0716	0.7866 ± 0.1084	0.756 ± 0.2	0.8714 ± 0.0937	0.8672 ± 0.112	0.8522 ± 0.098
	Fused	0.8282 ± 0.0778	0.8145 ± 0.0951	0.778 ± 0.1459	0.8778 ± 0.0864	0.879 ± 0.108	0.8717 ± 0.0822
**Vit (t-12)**	CQT	0.7519 ± 0.0866	0.7435 ± 0.0886	0.72 ± 0.105	0.7767 ± 0.0965	0.7825 ± 0.1198	0.8047 ± 0.1068
	Mel	0.7892 ± 0.0809	0.7686 ± 0.1147	0.738 ± 0.1834	0.8396 ± 0.084	0.8422 ± 0.1361	0.8564 ± 0.0959
	Gamma	0.8186 ± 0.0857	0.8 ± 0.1101	0.764 ± 0.1733	0.8651 ± 0.0638	0.8768 ± 0.0654	0.8547 ± 0.1036
	Fused	0.836 ± 0.0737	0.8282 ± 0.0813	0.806 ± 0.1343	0.8675 ± 0.0804	0.8677 ± 0.1009	0.8825 ± 0.0874
**Vit(b-16)**	CQT	0.6824 ± 0.0698	0.6872 ± 0.0974	0.726 ± 0.1731	0.6788 ± 0.0873	0.633 ± 0.133	0.7276 ± 0.0932
	Mel	0.7546 ± 0.0741	0.7499 ± 0.0875	0.754 ± 0.1357	0.7528 ± 0.0627	0.7555 ± 0.0601	0.8152 ± 0.091
	Gamma	0.7338 ± 0.0919	0.7142 ± 0.1171	0.7 ± 0.1925	0.7752 ± 0.1137	0.7733 ± 0.1602	0.7591 ± 0.1115
	Fused	0.7767 ± 0.0591	0.7546 ± 0.0796	0.704 ± 0.1236	0.8291 ± 0.0597	0.851 ± 0.0647	0.8219 ± 0.0716

Table S2. Performance comparison of different models with and without data augmentation.

Model	Augment	Accuracy	F1-Score	Sensitivity	Precision	Specificity	AUC
Eff.Net-B2	w/o augment	0.8168± 0.074	0.803 ± 0.091	0.770 ± 0.145	0.855 ± 0.084	0.866 ± 0.089	0.879 ± 0.087
	augment	0.843 ± 0.051	0.843 ± 0.055	0.850 ± 0.098	0.845 ± 0.066	0.838 ± 0.081	0.884 ± 0.073
DenseNet-121	w/o augment	0.8252 ± 0.08	0.8094 ± 0.098	0.7620 ± 0.139	0.8846 ± 0.096	0.8922 ± 0.102	0.8653 ± 0.094
	augment	0.842 ± 0.061	0.831 ± 0.077	0.806 ± 0.148	0.881 ± 0.075	0.883 ± 0.091	0.894 ± 0.063
ResNet-50	w/o augment	0.824 ± 0.0819	0.809 ± 0.097	0.772 ± 0.146	0.868 ± 0.077	0.879 ± 0.084	0.861 ± 0.085
	augment	0.837 ± 0.064	0.823 ± 0.072	0.768 ± 0.099	0.895 ± 0.067	0.908 ± 0.061	0.895 ± 0.074
ShuffleNet	w/o augment	0.772 ± 0.0881	0.752 ± 0.115	0.730 ± 0.180	0.8008 ± 0.094	0.816 ± 0.097	0.815 ± 0.109
	augment	0.800 ± 0.079	0.790 ± 0.089	0.774 ± 0.152	0.831 ± 0.104	0.829 ± 0.122	0.836 ± 0.0870
ViT (t)	w/o augment	0.831 ± 0.0791	0.813 ± 0.099	0.766 ± 0.147	0.888 ± 0.072	0.899 ± 0.073	0.879 ± 0.095
	augment	0.836 ± 0.0737	0.828 ± 0.081	0.806 ± 0.134	0.867 ± 0.080	0.867 ± 0.100	0.882 ± 0.0870

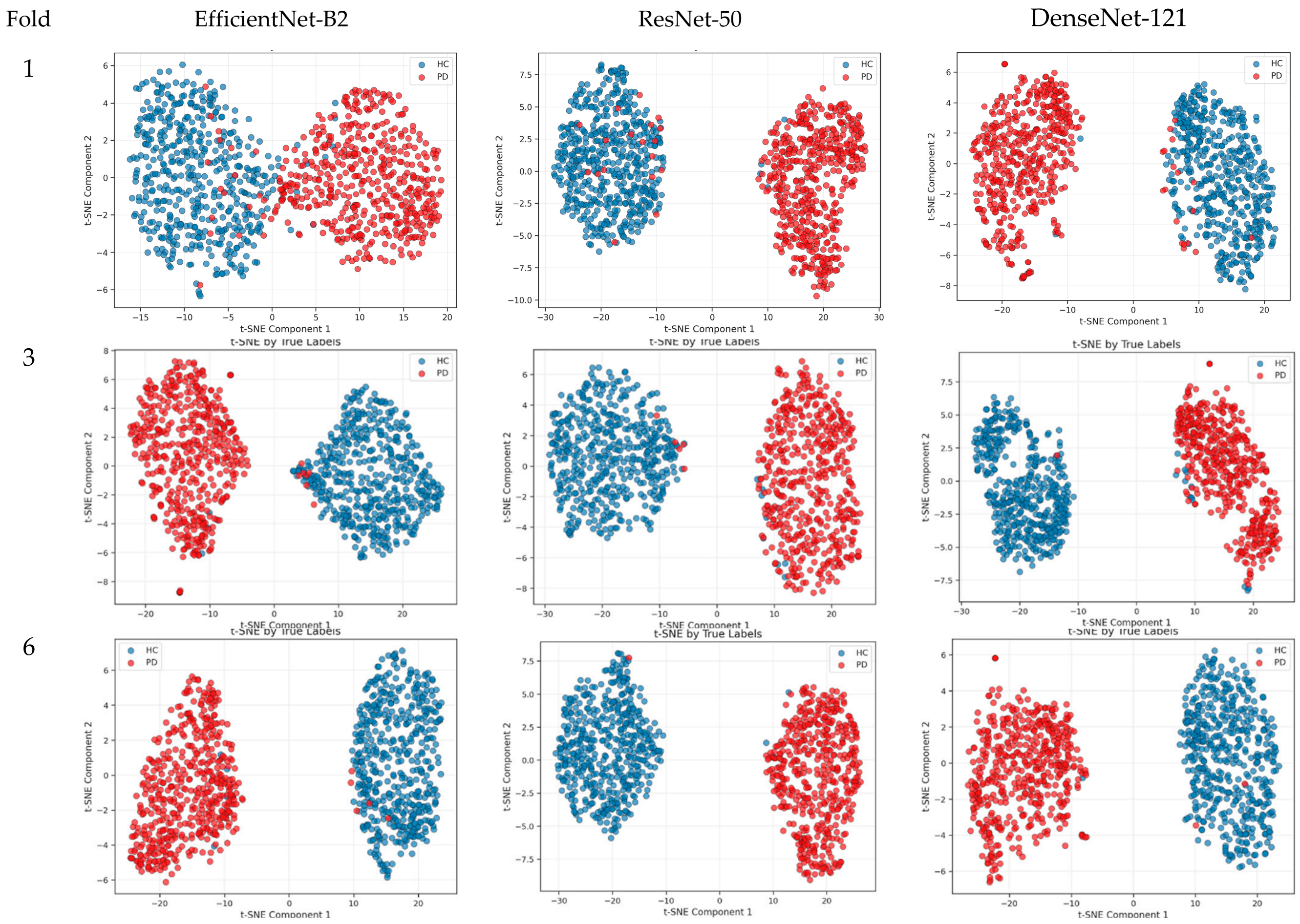

Figure S2. Comparison of t-SNE embeddings for HC and PD classes across three best-performing deep learning architectures across different folds: EfficientNet-B2, ResNet-50, and DenseNet-121, revealing distinct clustering patterns based on true labels.

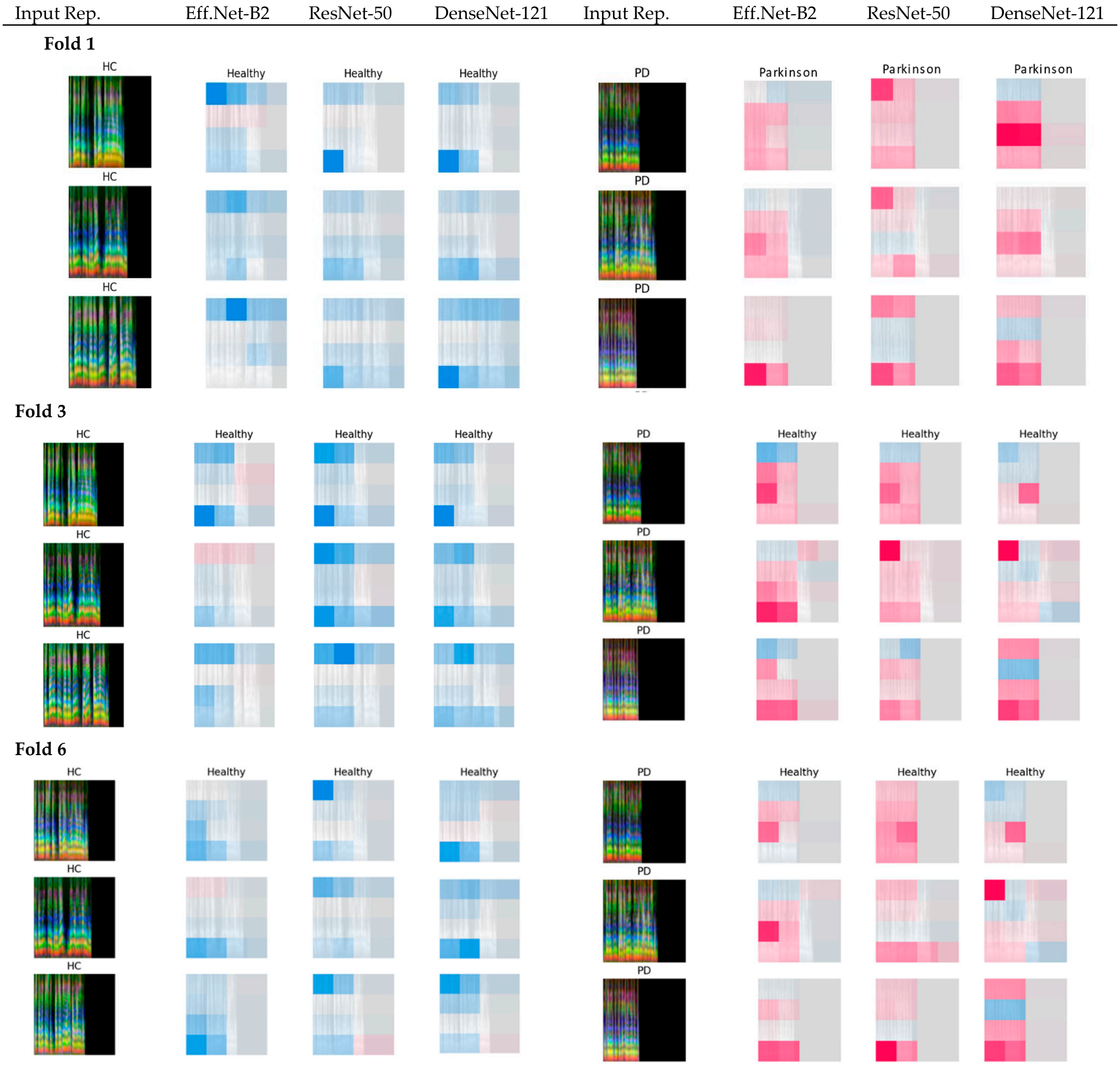

Figure S3. SHAP heatmaps across folds for PD detection using fused spectrograms. Left: fused spectrograms from Healthy Controls (HC) and Parkinson's Disease (PD) patients. Right: SHAP maps for EfficientNet-B2, ResNet-50, and DenseNet-121, with red indicating features driving "Parkinson" predictions and blue for "Healthy."

Reference

[1] J. R. Orozco-Arroyave and E. Noth, “New Spanish speech corpus database for the analysis of people suffering from Parkinson’s disease,” in *Proceedings of the Ninth International Conference on Language Resources and Evaluation (LREC’14)*, Reykjavik, Iceland: European Language Resources Association (ELRA), May 2014, pp. 342–347. [Online]. Available: http://www.lrec-conf.org/proceedings/lrec2014/pdf/7_Paper.pdf
